# The 12-month period prevalence and cardiac manifestations of HIV in patients with acute coronary syndrome at a tertiary hospital in Cape Town, South Africa: a retrospective cross-sectional study

**DOI:** 10.1186/s12879-021-06367-2

**Published:** 2021-07-07

**Authors:** Camilla Pennefather, Tonya Esterhuizen, Anton Doubell, Eric H. Decloedt

**Affiliations:** 1 Division of Clinical Pharmacology, Department of Medicine, Faculty of Medicine and Health Sciences, Stellenbosch University and Tygerberg Hospital, PO Box 241, Cape Town, 8000 Republic of South Africa; 2 Division of Epidemiology and Biostatistics, Department of Global Health, Faculty of Medicine and Health Sciences, Stellenbosch University, PO Box 241, Cape Town, 8000 Republic of South Africa; 3 Division of Cardiology, Department of Medicine, Faculty of Medicine and Health Sciences, Stellenbosch University and Tygerberg Hospital, PO Box 241, Cape Town, 8000 Republic of South Africa

**Keywords:** Acute coronary syndrome, HIV infection, Risk factors, Cardiac manifestations, Prevalence

## Abstract

**Background:**

HIV-positive patients are increasingly being affected by non-communicable diseases such as coronary artery disease (CAD). Data from high-income countries (HICs) indicate that HIV-positive patients have different risk-factor profiles for acute coronary syndrome (ACS) as well as different cardiac manifestations of this syndrome compared to HIV-negative patients. There is limited data from Sub-Saharan Africa (SSA), and particularly from South Africa with the biggest HIV epidemic in the world. The objective of this study was to determine the 12-month period prevalence of HIV in patients with ACS and to compare the risk-factor profile, ACS presentation and management between HIV-positive and HIV-negative adults.

**Methods:**

We included all patients hospitalised with ACS from 01 January to 31 December 2018 in a tertiary hospital, Tygerberg Hospital, in Cape Town, South Africa. The HIV-status of all patients was determined using routine clinical records. We performed multiple conditional logistic regression on HIV-positive and HIV-negative patients (1:3 ratio) to compare the risk factor profile, ACS presentation and management between the groups.

**Results:**

Among 889 patients, 30 (3.4%) were HIV-positive (95% confidence interval (CI): 2.3–4.8). HIV-positive patients were younger, more frequently men, and had a lower prevalence of medical comorbidities and a family history of CAD. They were more likely to present with ST-elevation myocardial infarction (STEMI) [odd’s ratio (OR) (95% CI): 3.12 (1.2–8.4)], and have single-vessel disease [OR (95% CI): 3.03 (1.2–8.0)]. Angiographic and echocardiographic data, as well as management, did not differ between the groups. Among HIV-positive patients, 17 (65%) were virally suppressed (HIV viral load < 200 copies/mL) with a median CD4^+^ count of 271 cells/mm^3^. The majority (20, 67%) of HIV-positive patients were receiving antiretroviral therapy at the time of the ACS.

**Conclusions:**

We found an HIV-prevalence of 3.4% (95% CI 2.3–4.8) in adults with ACS in a high endemic HIV region. HIV-positive patients were younger and more likely to present with STEMIs and single-vessel disease, but had fewer CAD risk factors, suggesting additional mechanisms for the development of ACS**.**

## Background

The incidence of acute coronary syndrome (ACS) in HIV-positive individuals is increasing [1]. Antiretroviral therapy (ART) treated HIV-positive patients are experiencing fewer HIV-related opportunistic infections and are increasingly being affected by the same age-associated spectrum of atherosclerotic disease, including coronary artery disease (CAD), as the general population [2]. Atherosclerotic disease, however, is emerging about 10 years earlier than in their uninfected counterparts, suggesting an alternative pathogenesis in HIV-positive patients [3]. CAD accounts for 8–22% of deaths among the HIV-positive population, making it an important cause of morbidity and mortality [4]. This is in contrast to the pre-ART era, when cardiomyopathies, pancarditis, conduction system abnormalities, pulmonary hypertension leading to heart failure, and neoplastic infiltration were more important cardiac manifestations of HIV infection [5].

Despite much data from Europe and North America, there are limited studies from South Africa, a low and middle-income country (LMIC), reporting on the prevalence of HIV in ACS, as well as the CAD risk factors, cardiac manifestations and management of ACS in these patients [6]. Most studies demonstrate HIV-positive patients with ACS to be younger, predominantly male, likely to smoke but with a lower prevalence of traditional CAD risk factors (diabetes mellitus, hypertension, dyslipidaemia) compared to their HIV-negative counterparts [1]. The increased incidence of ACS in the HIV-positive population suggests additional pathogenic mechanisms including increased survival time, ongoing HIV inflammation, immune-activation and ART-induced metabolic derangements [4, 7]. Chronic HIV-associated inflammation is associated with abnormal lipid profiles independent of ART, unstable plaque morphology, and more severe coronary artery stenosis [8]. Furthermore, it has been shown that HIV-positive patients have increased C-reactive protein, interleukin-6 and D-dimer levels, contributing to a pro-inflammatory and pro-thrombotic environment [5]. HIV-replication and immune-activation causes up-regulation of tissue factor pathways and chronic platelet activation, which may promote atherogenesis and put these patients at an increased risk of thrombotic events [7]. The clinical presentation of HIV-positive patients with CAD seems to be similar to that of the general population, and includes silent ischaemia, stable angina and ACS [7]. ACS is the main clinical presentation, of which ST-segment elevation myocardial infarction (STEMI) is the main sub-type. Non-ST-elevated myocardial infarction (NSTEMI) and unstable angina (UA) are the most common presentations of ACS in HIV-uninfected persons [1].

CAD is no longer a disease affecting just the high-income countries (HICs); LMICs today are experiencing a greater morbidity and mortality from ACS, with the death toll affecting the younger, productive ages more heavily [9]. This is evidenced by the shift from communicable diseases (48.1% prevalence in 2005; 31.3% prevalence in 2016) to non-communicable diseases (42.9% prevalence in 2005; 57.4% prevalence in 2016) as the primary cause of death in South Africans in the last decade [10]. Despite this, HIV still affects 13.1% of the South African population [11]. HIV-positive patients are hypothesised to be at an increased risk for CAD and the combined effect of these two distinct epidemics may have a marked impact on morbidity and life expectancy [7, 12].

We conducted a retrospective cross-sectional study to estimate the 12-month period prevalence of HIV in a South African population from Cape Town presenting with ACS. The second objective of the study aims to describe the CAD risk factors, cardiac manifestations, and management of HIV-positive patients in comparison to HIV-negative patients, using a case-control study design.

## Methods

We conducted a retrospective review of all adult patients presenting to Tygerberg Hospital (TBH), Cape Town, from 01 January to 31 December 2018 with ACS. TBH is the largest public-sector tertiary referral hospital in the Cape Town Metropole and serves over 3.4 million people; mostly vulnerable populations from densely populated low-income communities and rural areas.

Patients were identified using clopidogrel prescription data from the JAC electronic dispensing system [13]. We used the Electronic Content Management System (ECM) to access stored medical records to identify patients with the diagnosis of ACS documented in their medical record as the indication for clopidogrel treatment. The diagnosis of myocardial infarction (MI) at Tygerberg Hospital is made according to the Fourth Universal Definition: acute myocardial injury with clinical evidence of acute myocardial ischaemia and a rise of cardiac troponin levels with at least one value >99th percentile of the upper reference limit and at least one of the following: symptoms of myocardial ischaemia, new ischaemic changes on the ECG, pathological Q-waves on the ECG, imaging evidence of new loss of myocardium or new localised wall motion abnormality in a pattern indicative of ischaemic causes, presence of a coronary thrombus by angiography [14]. UA is diagnosed by the presence of ischaemic symptoms suggestive of ACS, without elevated biomarkers or ECG changes suggestive of infarction [14]. The diagnosis of ACS was confirmed by reviewing attending physician discharge summaries, and cross-checking the admission records, electrocardiograms (ECGs), troponin levels, echocardiograms and angiogram reports of each patient. Patients with repeated ACS-events within the year were only counted once, and their first ACS-event within the year was described.

We extracted the HIV status of all patients with a clinical diagnosis of ACS to determine the period prevalence. HIV data was obtained from the South African National Health Laboratory Service (NHLS) database. The NHLS is the only service provider of HIV testing in the public sector and an absent result implies that HIV testing was not done in the public sector. HIV-positive patients were classified as virally supressed (VL < 200 copies/mL) or virally unsuppressed (VL > 200 copies/mL), and the number of immune-suppressed patients (CD4^+^ count < 500 cells/mm^3^) was described.

**Fig. 1:**
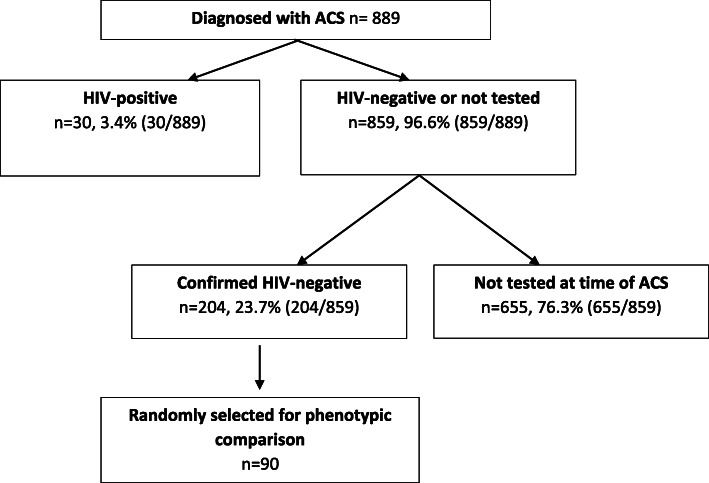
Diagram of the selection of patients for study inclusion

In the case-control analysis of our study we compared the CAD risk factors, ACS-type and management of HIV-positive compared with HIV-negative patients with ACS. In order to do this, we selected three HIV-negative patients (confirmed HIV-negative test result on the NHLS) for every HIV-positive patient (1:3 ratio) (Fig. 1). The HIV-negative patients were chosen by sequentially selecting the next three patients with a negative HIV-test following the HIV-positive patient, by date order of clopidogrel dispensing. Our method did not allow for matching according to baseline characteristics. We extracted the following CAD variables from their medical records on the ECM system: comorbidities (hypertension, dyslipidaemia, diabetes mellitus), family history of CAD, social risk factors (tobacco-smoking, body mass index > 25 kg/m^2^, ethanol-misuse), the type of ACS (STEMI, NSTEMI and UA), the culprit coronary artery, the region of myocardium involved, single- or multi-vessel coronary artery disease, echocardiographic findings, pharmacological and interventional management of the patients, as well as their serum creatinine concentration on presentation. We identified medical comorbidities using discharge summary diagnoses, prescription data and medical records on the ECM. In the public sector, hypertension, diabetes mellitus and dyslipidaemia are diagnosed and managed according to the Adult Hospital Level Standard Treatment Guidelines and Essential Medicines List [15]. Diagnosis of hypertension requires at least 3 blood pressure readings ≥140/90 at least 2 days apart. Diagnosis of diabetes mellitus is made based on the following: symptoms of hyperglycaemia or metabolic decompensation with any one single test that confirms a random plasma glucose ≥11.1 mmol/L, fasting plasma glucose ≥7.0 mmol/L, HbA1c ≥ 6.5% or 2-h post-load glucose ≥11.1 mmol/L. Dyslipidaemia is diagnosed based on the finding of abnormal serum lipid levels, and treatment thereof is determined by a cardiovascular risk score (taking into account total cholesterol, high-density lipoprotein (HDL), gender, age and smoking status) [15].

The calculation of the 12-month period prevalence of HIV-positive patients presenting to TBH with ACS in 2018 was determined from the entire 2018 sample using a simple proportion calculation. From this sample, a sub-sample consisting of just those infected with HIV (exposed) and three times the number of exposed with a confirmed negative HIV test (unexposed) were selected for further statistical analysis. Data were analysed using Stata version 11.0 (StatCorp, College Station, TX, USA). Baseline characteristics of exposed and unexposed patients were compared by chi-square tests or 2-sided Fisher’s exact tests for categorical variables and T-tests or Wilcoxon rank-sum tests for continuous variables. Normally distributed data were described using means, medians and standard deviations (SD). 95% confidence intervals (CIs), odds ratios (ORs), and *p*-values were derived for all appropriate data. Logistic regression was performed, controlling for age and sex, in order to determine associations between risk factor profile/cardiac manifestations in those presenting with ACS and HIV-status. Adjusted 95% CIs, ORs and *p*-values were derived after such adjustment. Statistical significance was set at a *p*-value of less than 0.05.

Ethical approval for the study as well as a waiver of consent was obtained from the Human Research Ethics Committee of the Faculty of Medicine and Health Sciences of Stellenbosch University (Reference number: U19/05/023).

## Results

### Prevalence of HIV in ACS

We identified 889 patients who presented to TBH in 2018 with the diagnosis of ACS of which 30 patients were HIV-positive to give a 12 month period prevalence of 3.4% (95% CI 2.3–4.8) (Fig. 1).

### Baseline characteristics

Thirty HIV-positive ACS patients were compared with 90 randomly selected HIV-negative ACS patients, with the following findings (Table 1): The HIV-positive patients were on average 7 years younger (50 years compared with 57 years), more frequently men (*n* = 20/30; 67% compared with *n* = 50/90; 56%), had a lower prevalence of medical comorbidities (hypertension, dyslipidaemia and diabetes mellitus) (*n* = 20/30; 67% compared with *n* = 83/90; 92%), and a lower prevalence of a family history of CAD (*n* = 2/30; 7% compared with *n* = 18/90; 20%), even after adjusting for age and sex. There was no difference in the social risk factor profile of the patients between the two groups. Although statistically nonsignificant, mention is made of the 11 μmol/L higher median serum creatinine in the HIV-negative sub-group.

**Table 1:** Characteristics of HIV-positive compared to HIV-negative patients (crude and adjusted)

Characteristic, n (%) except where specified	HIV+ (*n* = 30)	HIV- (*n* = 90)	Crude OR (95% CI)	^a^aOR (95% CI)	adjusted *p*-value
Demographics					
Age (years), mean (SD)	**50 (11.90)**	**57.32 (11.63)**	**0.95 (0.91–0.98)**		**0.008**^#^
Male	20 (66.7%)	50 (55.6%)	1.60 (0.67–3.80)		0.586^#^
Cardiovascular risk factors					
Medical comorbidities	**20 (66.7%)**	**83 (92.2%)**	**0.17 (0.06–0.50)**	**0.21 (0.07–0.65)**	**0.007**
Hypertension	**13 (43.3%)**	**64 (71.1%)**	**0.31 (0.13–0.73)**	**0.39 (0.16–0.97)**	**0.044**
Dyslipidaemia	**7 (23.3%)**	**45 (50.0%)**	**0.30 (0.12–0.78)**	**0.28 (0.11–0.76)**	**0.012**
Diabetes mellitus	4 (13.3%)	34 (37.8%)	0.25 (0.08–0.79)	0.31 (0.10–1.00)	0.051
Personal history CAD	4 (13.3%)	27 (30.0%)	0.36 (0.11–1.13)	0.48 (0.15–1.60)	0.234
Personal history CVA	3 (10.0%)	10 (11.1%)	0.89 (0.23–3.47)	0.70 (0.17–2.93)	0.630
PVD	3 (10.0%)	10 (11.1%)	0.89 (0.23–3.47)	1.08 (0.26–4.51)	0.913
Family history CAD	**2 (6.7%)**	**18 (20.0%)**	**0.29 (0.06–1.31)**	**0.20 (0.04–0.99)**	**0.048**
Social risk factors	25 (83.3%)	74 (82.2%)	1.08 (0.36–3.25)	0.42 (0.11–1.55)	0.192
Smoking	23 (76.7%)	67 (74.4%)	1.13 (0.43–2.97)	0.57 (0.18–1.76)	0.329
BMI > 25 kg/m^2^	7 (23.3%)	29 (32.2%)	0.64 (0.25–1.66)	0.56 (0.21–1.50)	0.249
Ethanol-misuse	3 (10.0%)	10 (11.1%)	0.89 (0.23–3.47)	0.60 (0.14–2.45)	0.472
Serum creatinine (μmol/L), median (IQR)	71.5 (59–98)	82.5 (66–103)	0.99 (0.97–1.00)	0.99 (0.98–1.01)	0.361

*OR* odds ratio, *CI* confidence interval, *SD* standard deviation, *CAD* coronary artery disease, *CVA* cerebrovascular accident, *PVD* peripheral vascular disease, *BMI* body mass index, *IQR* inter-quartile range

^a^adjusted odds ratio conditioned on age and sex. ^#^crude *p*-value. Bolded text = statistically significant

### Acute coronary syndrome information

Twenty-three (*n* = 23/30; 76.6%) HIV-positive patients presented with a STEMI compared to 48 (*n* = 48/90; 53.3%) controls, with HIV-positive patients having 3.12 greater odds (95% CI 1.2–8.4) of presenting with a STEMI after adjusting for age and sex (Table 2). Furthermore, HIV-positive patients had 3.03 greater odds (95% CI 1.2–8.0) of having single-vessel disease after adjusting as above (*n* = 16/30; 53% compared with *n* = 19/90; 21%). Although statistically nonsignificant, mention can be made of the increased prevalence of left anterior descending (LAD) artery involvement in the HIV-positive group (*n* = 16/30; 53.3%) compared to the HIV-negative group (*n* = 38/90; 42.2%), translating into more LAD-territory infarcts in the former (Table 3).

**Table 2:** ACS presentation of HIV-positive compared to HIV-negative patients (crude and adjusted)

Outcome, n (%)	HIV+ (*n* = 30)	HIV- (*n* = 90)	Crude OR (95% CI)	^a^aOR (95% CI)	Adjusted *p*-value
ACS type					
STEMI	**23 (76.7%)**	**48 (53.3%)**	**2.88 (1.12–7.37)**	**3.12 (1.16–8.40)**	**0.024**
NSTEMI (reference)	7 (23.3%)	32 (35.6%)			
UA (reference)	0 (0.00)	10 (8.3%)			
Single/multi-vessel disease					
Single-vessel	**16 (53.3%)**	**19 (21.1%)**	**4.27 (1.78–10.27)**	**3.03 (1.16–7.96)**	**0.024**
Multi-vessel (reference)	14 (46.7%)	71 (78.9%)			

^a^adjusted odds ratio conditioned on age and sex. Bolded text = statistically significant. *OR* odds ratio, *CI* confidence interval, *ACS* acute coronary syndrome, *STEMI* ST-elevation myocardial infarction, *NSTEMI* non-ST-elevation myocardial infarction, *UA* unstable angina

**Table 3:** Angiographic data and management of HIV-positive compared to HIV-negative patients

Outcome, n (%)	HIV+ (*n* = 30)	HIV- (*n* = 90)	*p*-value
Culprit coronary artery			
Left main stem	0 (0.0%)	2 (2.2%)	1
Left anterior descending	16 (53.3%)	38 (42.2%)	0.299
Diagonal (1st branch)	1 (3.3%)	5 (5.6%)	1.000
Circumflex	4 (13.3%)	8 (8.9%)	0.492
Oblique marginal (1st branch)	1 (3.3%)	3 (3.3%)	1.000
Right coronary artery	6 (20.0%)	25 (27.8%)	0.476
Other	1 (3.3%)	1 (1.1%)	0.439
Region affected by STEMI			
Antero-septal	13 (56.5%)	26 (54.2%)	1
Lateral	9 (39.1%)	11 (22.9%)	0.171
High-lateral	1 (4.4%)	4 (8.3%)	1
Inferior	9 (39.1%)	22 (45.8%)	0.620
Posterior	2 (8.7%)	8 (16.7%)	0.482
Right ventricle	1 (4.4%)	2 (4.2%)	1
Other	0 (0.0%)	1 (2.1%)	1
Radiological investigations			
Echocardiogram	22 (73.3%)	77 (85.6%)	0.165
MRI	1 (3.3%)	6 (6.7%)	0.670
Intervention in those with STEMI			
Thrombolysis	17 (73.9%)	30 (62.5%)	0.341
PCI	16 (69.6%)	38 (79.2%)	0.375
Conservative	2 (8.7%)	5 (10.4)	0.820
Discharge medication			
Antiplatelet	30 (100.0%)	90 (100.0%)	1
Angiotensin converting enzyme-inhibitor	26 (86.7%)	79 (87.8%)	1
Angiotensin receptor blocker	1 (3.3%)	7 (7.8%)	0.678
Beta-blocker	30 (100.0%)	86 (95.6%)	0.571
Statin	28 (93.3%)	90 (100.0%)	0.061
Simvastatin	25 (92.6%)	88 (97.8%)	0.227
Atorvastatin	2 (7.4%)	2 (2.2%)	0.227
Nitrate	2 (6.7%)	9 (10.0%)	0.729
Calcium-channel blocker	0 (0.0%)	6 (6.7%)	0.335
Thiazide diuretic	0 (0.0%)	7 (7.8%)	0.190
Loop diuretic	5 (16.7%)	17 (18.9%)	1
Potassium-sparing diuretic	1 (3.3%)	1 (1.1%)	0.439

*STEMI* ST-elevation myocardial infarction, *MRI* magnetic resonance imaging, *PCI* percutaneous coronary intervention

### Echocardiographic information

There was no difference in the echocardiographic data between the two groups, with the exception of the left ventricular internal dimension at end-systole (LVIDs), which was statistically significantly smaller in the HIV-positive compared to the HIV-negative sub-group (3.37 mm compared with 3.96 mm) (Table 4). The systolic function was equally impaired in both groups.

**Table 4:** Echocardiographic parameters of HIV-positive compared to HIV-negative patients

Variable, mean (SD) except where specified	HIV+	HIV-	*p*-value
LA diameter (mm) (*n* = 48)	3.47 (0.52)	3.84 (0.67)	0.124
LA area (cm^2^) (*n* = 73)	17.89 (4.28)	19.58 (4.80)	0.219
LVIDs (mm) (*n* = 73)	**3.37 (0.54)**	**3.96 (0.90)**	**0.019**
LVIDd (mm) (*n* = 77)	4.72 (0.49)	5.11 (0.83)	0.083
Ejection fraction (%) (*n* = 86)	45.4% (11.56)	44.3% (12.46)	0.758
E/e’ (*n* = 69)	12.18 (5.06)	14.06 (8.22)	0.4171
Effusion: n (%) (*n* = 77)	2 (13.3%)	7 (11.3%)	1

Bolded text = statistically significant. *SD* standard deviation, *LA* left atrium, *LVIDs* left ventricular internal diameter end-systole, *LVIDd* left ventricular internal dimension end-diastole, *E/e’* ratio between early mitral inflow velocity and mitral annular early diastolic velocity

### HIV-specific information

The median CD4^+^ count in those with available CD4^+^ results in the HIV-positive sub-group was 271 cells/mm^3^ (Table 5). Twenty-two patients (*n* = 22/27; 81.5%) had a CD4^+^ count <500cells/mm^3^, with a nadir CD4^+^ count of 41 cells/mm^3^. Seventeen (*n* = 17/26; 65.4%) patients were virally suppressed (HIV VL < 200 copies/mL) and 13 (*n* = 13/26; 50%) had viral loads lower than detectable limits (HIV VL < 20 copies/mL). The majority of the HIV-positive patients were on ART at the time of the ACS event (*n* = 20/30; 66.7%), while eight (*n* = 8/30; 26.7%) were ART-naïve and two (*n* = 2/30; 6.7%) had defaulted their medication at the time of the event. Of the HIV-positive patients receiving ART, 15 (*n* = 15/20; 75%) were on a non-nucleoside reverse transcriptase inhibitor (NNRTI) – based regimen and five (*n* = 5/20; 25%) were on a protease inhibitor (PI) – based regimen.

**Table 5:** Immunological and ART status of HIV-positive patients

Variable, n (%) except where specified	HIV+
Viral load (copies/mL) (*n* = 26)	
Suppressed (< 200)	17 (65.4%)
Unsuppressed (> 200)	9 (34.6%)
CD4^+^ count (cells/mm^3^) (*n* = 27)	
Median (IQR)	271 (355)
ART status (*n* = 30)	
Treated with ART at time of event	20 (66.7%)
ART-naïve	8 (26.7%)
Defaulted ARTs	2 (6.7%)
ART regimen (*n* = 20)	
Tenofovir-emtricitibine-efavirenz	14 (70.0%)
Abacavir-lamivudine-efavirenz	1 (5.0%)
Zidovudine-lamivudine-lopinavir/ritonavir	4 (20.0%)
Stavudine-lamivudine-lopinavir/ritonavir	1 (5.0%)

*IQR* inter-quartile range, *ART* anti-retroviral therapy

### Management

There was no difference in the immediate or long-term management of the patients (Table 3). Rates of thrombolysis in patients with STEMIs did not differ significantly between HIV-positive and HIV-negative patients.

## Discussion

In this retrospective cross-sectional analysis of patient records at a large tertiary hospital in Cape Town, South Africa, the 12-month period prevalence of HIV in those hospitalised with ACS was 3.4% (95% CI 2.3–4.8). HIV-positive patients were younger, predominantly male, had a lower prevalence of medical comorbidities and a lower prevalence of a family history of CAD, even after adjusting for age and sex. HIV-positive patients experienced more STEMIs and single-vessel disease. Our findings contribute data on the prevalence and presentation of HIV in ACS from South Africa with the largest ART program in the world and a rising rate of non-communicable disease-attributed morbidity and mortality [16].

The relative absence of traditional risk factors in the HIV-positive sub-group suggests additional pathogenic mechanisms in these patients; one being the higher rates of sub-clinical dyslipidaemia seen in HIV-positive individuals. HIV autopsy studies have shown evidence of premature CAD in HIV-positive patients even before initiation of ART, as a result of complex dyslipidaemic patterns: reduced total serum cholesterol, reduced high-density lipoprotein (HDL), reduced apolipoprotein B, and increased low-density lipoprotein (LDL) [4]. Despite a lower prevalence of diagnosed dyslipidaemia in our HIV-positive sub-group, we do not have data on their lipid profiles at the time of presentation for ACS, which may have revealed a higher prevalence of sub-clinical dyslipidaemia. We did not find an association between HIV-positive patients suffering from ACS and those receiving PIs which makes it plausible that there is another risk factor apart from PIs in the causation of dyslipidaemia and ACS. Our sample size is however limited to draw definitive conclusions. The cardiovascular protective effect of ART however is supported by early ART-initiation to prevent CAD in HIV-positive individuals, as well as the increased risk of CAD and other comorbidities associated with ART-interruption [17]. Therefore, it is plausible that it is the combined effect of HIV infection and certain ART drugs that results in complex sub-clinical dyslipidaemic patterns in HIV-positive patients, thereby increasing their risk for CAD and resultant ACS.

An additional suggested pathogenic mechanism for CAD in HIV-positive individuals is immune-system dysfunction, which can be directly measured by the number of CD4^+^ lymphocytes in the body. Lichtenstein et al. showed that a CD4^+^ count of less than 500 cells/mm^3^ is an independent risk factor for CAD, with comparative attributable risk of approximately 20% - a figure similar to several other traditional CAD risk factors [18]. The majority (81%) of patients in our study had CD4^+^ counts less than 500cells/mm^3^ which suggests an increased risk of CAD in these patients. Current detectable viraemia has been found to be a further risk factor for CAD, due to its contribution to an environment of persistent chronic inflammation [17]. Even virally suppressed HIV-positive patients have higher levels of inflammatory markers than those without HIV; thereby predisposing them to CAD [8].

The predominant presentation of single-vessel CAD and STEMIs in the HIV-positive patients in our study (even after adjusting for age and sex) is most likely due to the unique histological characteristics of coronary plaques in these patients. Virtual histology intravascular ultrasound analysis of HIV-positive patients affected by ACS has shown a high prevalence of unstable plaque morphology that is rich in necrotic tissue, less calcific, and has a thicker fibrous cap compared to that seen in traditional CAD [4]. Non-calcified plaques are more likely to rupture than calcific or mixed plaques, putting these patients at higher risk of single-vessel STEMI than their HIV-negative counterparts [19]. This higher plaque vulnerability is thought to be linked to the chronic inflammatory process of being infected with the HI virus itself. Furthermore, Moran et al. found that HIV-positive patients with single-vessel disease had higher Gensini Scores [20] than HIV-negative controls with single-vessel disease, indicating more severe vessel stenosis in the former [19]. The presence of more extensive, more vulnerable non-calcific, fibro-fatty plaque could also explain the higher prevalence and earlier onset of ACS in the HIV-positive population.

The mild systolic dysfunction seen in both groups is in keeping with the echocardiographic changes expected after a myocardial infarction. We would expect more advanced diastolic dysfunction in our HIV-negative sub-group as a result of the increased prevalence of hypertension in this group. A study done in Cameroon comparing the left atrial remodelling in hypertensives compared to healthy patients showed the hypertensive patients to have a larger left atrial diameter, surface area and volume, indicating an altered diastolic function in these patients [21]. Although statistically nonsignificant, it is plausible that the 11 μmol/L higher mean serum creatinine in the HIV-negative sub-group can be explained by the increased prevalence of medical comorbidities in this group, resulting in a higher incidence of target-organ damage which may manifest as sub-clinical/clinical chronic kidney disease. Serum creatinine is reported in many studies as a prognostic marker for overall cardiac mortality. In a study by Matts et al. it was found that each nine μmol/L (0.1 mg/dL) rise in baseline serum creatinine had a 36% increased relative risk of future overall mortality and a 47% increased relative risk for future atherosclerotic CAD mortality (no confounding factors present) [22]. This potentially translates into a 44% increased relative risk of overall future mortality and a 57% increased relative risk for future atherosclerotic CAD mortality in the HIV-negative patients in our study. Such a finding in our study cannot be over-interpreted due to our small sample size.

As there is no current evidence for a change in immediate or long-term management of CAD and ACS based on HIV status, all patients in our study were treated similarly. Simvastatin coadministered with PIs are expected in increase simvastatin concentrations markedly due to CYP3A inhibition [23]. However, we noted that 80% of patients on PIs incorrectly received simvastatin. The more frequent prescription of calcium-channel blockers and thiazide diuretics in the HIV-negative sub-group is most likely as a result of the increased prevalence of hypertension in this population.

Overall, our findings were consistent with the literature. There is limited global data on the prevalence of HIV in people presenting with ACS, but our findings are similar to the 2.4% HIV prevalence found in the CAD sub-group (consisting of 581 patients) of all de novo cases of heart disease presenting to a tertiary hospital in Soweto, South Africa [24]. These values are significantly lower than both the 13.1% estimated national HIV-prevalence in South Africa in 2018 and the 7% prevalence of HIV in a population of people with ACS in a HIC such as Spain [11]. This may be due to the under-reporting of HIV in ACS in South Africa as evidenced by the large portion of untested patients in our study (Fig. 1) [1]. Furthermore, it may be due to other confounding factors; one being age: HIV-prevalence is highest in young adults whereas ACS primarily affects the elderly. Despite this, data from a study conducted in Québec, Eastern Canada, showed the incidence of ACS to be 3.88 in the HIV-positive cohort compared to 2.21 in the HIV-negative cohort per 1000 patient-years, irrespective of exposure to ART [25]. A study conducted in Boston, Massachusetts, showed increased ACS rates per 1000 person-years in HIV-positive vs HIV-negative patients (11.13 vs 6.98), even after adjusting for age, sex, race, hypertension, diabetes and dyslipidaemia [12]. The demographic profile and CAD risk factor profile of the patients in our study was consistent with that seen in the literature. Furthermore, the type of ACS and number of involved coronary arteries of the HIV-positive patients in our study matched that seen in the literature. Various studies from the USA and France showed angiographic findings of fewer involved vessels and a greater burden of inflammatory plaque in the HIV-positive cases as compared to the HIV-uninfected cases [7]. Our findings of a predilection for LAD artery involvement in the HIV-positive patients are supported by those of Vachiat et al. who showed the LAD to be the most common culprit artery (60%) in HIV-positive patients with ACS [26]. The same proportion of HIV-positive and HIV-negative patients in our study underwent percutaneous coronary intervention (PCI), but we have no follow-up data on long-term success rates of such an intervention. A Spanish study showed a lower long-term success-rate in HIV-positive patients who underwent PCI compared to HIV-negative patients (75% versus 85% success-rate respectively) [27].

An interesting finding from our study was that despite a nonsignificant increased unadjusted prevalence of smoking seen in HIV-positive compared to HIV-negative patients, this finding was reversed when adjusting for age and sex. This finding could perhaps explain the heterogeneity in smoking data when comparing HIV-positive and HIV-negative patients with ACS. The majority of studies show a positive correlation between HIV-infection and smoking; however, when matching on age and sex, Dwyer et al found no difference in rates of smoking between HIV-positive and HIV-negative sub-groups [1, 28].

Our study has a number of limitations. First, the retrospective, observational design limits its ability to control for unmeasured confounders. Second, our sample size is limited which did not allow us to perform sub-group analyses. Third, we may have underestimated the HIV-prevalence within the ACS population due to the relative infrequent reporting of HIV-results. It is not routine for everyone presenting to a healthcare facility in South Africa to be tested for HIV, meaning that the actual prevalence of HIV within the ACS population might be higher than reported in our study. Fourth, our sample is from a single centre and may not be representative of South Africa or sub-Saharan Africa. Last, we were confronted with missing data that were not always collected as part of routine clinical care. CD4^+^ counts and viral loads were not available for all HIV-positive patients, and when available, were not always taken during the same admission as for the ACS event. It is also possible that we may have missed cases with our retrospective design. However, given that we used electronic database systems for our case identification and data extraction, we argue that this risk was minimised.

## Conclusions

In conclusion, we found that HIV-positive patients with ACS were younger, more likely to present with STEMIs and single-vessel disease, and had limited traditional ACS risk factors. This suggests additional pathogenic mechanisms for the development of CAD and ACS in patients with HIV; possibly attributed to the inflammation and immune-activation caused by infection with HIV. To date, ART is the most important treatment intervention highlighting the importance of prompt initiation of ART in patients with HIV [29]. The younger age of ACS presentation in patients with HIV highlights the importance of early, rigorous screening for risk factors and early diagnosis of CAD for active management. Our prevalence finding of 3.4% of HIV in ACS may be an under-estimate in South Africa and should be prospectively studied.

## Data Availability

The datasets used and/or analysed during the current study are available from the corresponding author on reasonable request.

## Abbreviations

CAD: Coronary artery disease
HIC: High-income country
ACS: Acute coronary syndrome
TBH: Tygerberg Hospital
SSA: Sub-Saharan Africa
CI: Confidence interval
STEMI: ST-elevation myocardial infarction
OR: Odd’s ratio
ART: Anti-retroviral therapy
LMIC: Low/middle-income country
NSTEMI: Non-ST-elevation myocardial infarction
UA: Unstable angina
ECG: Electrocardiogram
ECM: Electronic Content Management System
MI: Myocardial infarction
NHLS: National Health Laboratory Service
SD: Standard deviation
LAD: Left anterior descending
LVIDs: Left ventricular internal dimension at end-systole
VL: Viral load
NNRTI: Non-nucleoside reverse transcriptase inhibitor
PI: Protease inhibitor
HDL: High-density lipoprotein
LDL: Low-density lipoprotein
PCI: Percutaneous coronary intervention
